# NIR and Py-mbms coupled with multivariate data analysis as a high-throughput biomass characterization technique: a review

**DOI:** 10.3389/fpls.2014.00388

**Published:** 2014-08-07

**Authors:** Li Xiao, Hui Wei, Michael E. Himmel, Hasan Jameel, Stephen S. Kelley

**Affiliations:** ^1^Department of Forest Biomaterials, North Carolina State UniversityRaleigh, NC, USA; ^2^National Renewable Energy Laboratory, Biosciences CenterGolden, CO, USA

**Keywords:** biomass characterization, lignocellulosic biofuel, near infrared spectroscopy, pyrolysis molecular beam, mass spectrometry, multivariate data analysis, high throughput, chemometrics

## Abstract

Optimizing the use of lignocellulosic biomass as the feedstock for renewable energy production is currently being developed globally. Biomass is a complex mixture of cellulose, hemicelluloses, lignins, extractives, and proteins; as well as inorganic salts. Cell wall compositional analysis for biomass characterization is laborious and time consuming. In order to characterize biomass fast and efficiently, several high through-put technologies have been successfully developed. Among them, near infrared spectroscopy (NIR) and pyrolysis-molecular beam mass spectrometry (Py-mbms) are complementary tools and capable of evaluating a large number of raw or modified biomass in a short period of time. NIR shows vibrations associated with specific chemical structures whereas Py-mbms depicts the full range of fragments from the decomposition of biomass. Both NIR vibrations and Py-mbms peaks are assigned to possible chemical functional groups and molecular structures. They provide complementary information of chemical insight of biomaterials. However, it is challenging to interpret the informative results because of the large amount of overlapping bands or decomposition fragments contained in the spectra. In order to improve the efficiency of data analysis, multivariate analysis tools have been adapted to define the significant correlations among data variables, so that the large number of bands/peaks could be replaced by a small number of reconstructed variables representing original variation. Reconstructed data variables are used for sample comparison (principal component analysis) and for building regression models (partial least square regression) between biomass chemical structures and properties of interests. In this review, the important biomass chemical structures measured by NIR and Py-mbms are summarized. The advantages and disadvantages of conventional data analysis methods and multivariate data analysis methods are introduced, compared and evaluated. This review aims to serve as a guide for choosing the most effective data analysis methods for NIR and Py-mbms characterization of biomass.

## INTRODUCTION FOR BIOMASS CHEMICAL COMPOSITION

Biomass is a complicated mixture of organic and inorganic compounds. It is mainly composed of cellulose, hemicelluloses and lignins, as well as minor components, such as proteins, extractives, ash, and other nonstructural mineral materials. Because of its renewable nature and chemical composition, biomass is an attractive feedstock for energy and chemical products (Ragauskas et al., 2006; Himmel et al., 2007; Wei et al., 2009; Sluiter et al., 2010). In order to provide an effective guide for feedstock selection and process development, it is very important to measure biomass chemical composition accurately and efficiently (Sluiter et al., 2010; Templeton et al., 2010; Daystar et al., 2013). In this paper, we will review the use of two high-throughput techniques, near infrared spectroscopy (NIR) and pyrolysis-molecular beam mass spectrometry (Py-mbms) in biomass characterization. The advantages and disadvantages of different data analysis methods, including band/peak assignment, tools for spectral treatments and resolution enhancement and multivariate data analysis methods, are introduced, compared and evaluated. Selected research publications are reviewed and categorized as “case studies” according to the ways they analyzed data and the specific biomass properties that are evaluated.

## CONVENTIONAL BIOMASS CHARACTERIZATION RELEVANT TO BIOFUEL PRODUCTION

Traditional biomass compositional analysis, based on two-stage sulfuric acid hydrolysis followed by gravimetric and instrumental analysis, has been used to measure lignin and carbohydrates for more than 100 years. These methods have been used by researchers for studies of wood materials, animal food, human health, bioenergy production, and many other areas related to biomaterials. The history and uses of these methods were reviewed in detail elsewhere (Sluiter et al., 2010). The analytical uncertainty for different methods was also evaluated by statistical analysis and reported as the standard deviation of measurement for each component (Templeton et al., 2010). Other wet chemical techniques also include: acidolysis, thioacidolysis, nitrobenzene oxidation, transesterification, acetyl bromide method, orcinol method, Van Soest method, etc. Routine procedures, a number of less common methods, and new analytical methods developed for research purposes in the field of wood chemistry are described in books (Browning, 1967; Sjöström and Alén, 1999). These techniques quantify important chemical structure biomass, but they are time consuming and laborious.

Separately, combustion-related properties are of interest for the utilization of biomass in biofuel and biopower production. There are three types of combustion-related properties: morphological, physical, and chemical properties (Braadbaart and Poole, 2008). Traditional fuel analysis of biomass includes ultimate analysis, proximate analysis, and thermogravimetric analysis. In addition, ash composition and sulfur can be determined and used to predict fuel indices, especially for slagging behavior, aerosol formation, and corrosion related risks (Obernberger, 2014).

## USE OF SPECTROSCOPIC TOOLS IN BIOMASS CHARACTERIZATION AS HIGH THROUGHPUT TECHNIQUES

Spectroscopic methods, such as Fourier transform infrared spectroscopy (FTIR), NIR, Raman spectroscopy (Raman), and nuclear magnetic resonance (NMR), are widely used to measure functional groups and chemical bonds in biomass. These measurements are faster and more convenient than most conventional chemical methods used for biomass characterization and fuel analysis. Besides, since there is no degradative chemical treatment used during analysis, the information gained from these tools is more representative of the chemical structures in original biomass. However, there are some drawbacks for using these spectroscopic tools. For example, data interpretation for FTIR, Raman, and NMR is relatively complicated, sample preparation can be complex, and due to the mixed nature of biomass, peak assignment usually suffers from the overlap of many compounds. A good summary of spectroscopic tools used as high throughput techniques in biomass study can be found in a recent review (Lupoi et al., 2014).

## HIGH THROUGHPUT TECHNIQUES COUPLED WITH MULTIVARIATE STATISTICAL ANALYSIS

Because of many chemical features included in a single spectrum, it is challenging to elucidate data directly for a group of samples. Therefore, multivariate analysis (MVA) tools have been widely used in spectroscopic data analysis (Jin and Xu, 2011; Smith-Moritz et al., 2011; Xu et al., 2013; Lupoi et al., 2014). Among them, the two multivariate tools that have been widely used are: (1) Principal component analysis (PCA), and (2) Partial least square (PLS).

Principal component analysis is mainly used for identifying outliers, sample comparison, and screening. It relies on projecting original samples variables on several (usually <six) reconstructed variables which are representative of original sample variation. Those reconstructed variables are known as principal components (PCs). Samples described with PCs can be plotted in scores plot, in which similar samples cluster together while samples different from each other are separated in two-, three-, or n-dimensional coordinates. Together with the scores plot, PCA loadings plot allows for the determination of important chemical features responsible for the sample grouping. In the loadings plot, variables with large values are highly correlated with sample grouping (Sykes et al., 2009).

Partial Least Square is used to build prediction correlation models between spectral data and the property of interest. In the application of NIR and Py-mbms, spectral data is regarded as “predictors” for the biomass properties of interest. The properties of a new sample can then be estimated using a PLS model built from spectral data taken on a set of similar samples with known characteristics. In this way, time consuming experiments for new samples could be eliminated. Regression coefficients are generated and can be used to relate chemical features in the spectra to the specific sample properties (Labbe et al., 2006).

In summary, multivariate tools used in spectroscopic data analysis have three functions: (1) comparing sample similarities and differences and discovering outliers; (2) building prediction models between spectroscopic data and biomass properties of interest; and (3) discovering correlations between property data and spectral data.

## BIOMASS CHARACTERIZATION BY NIR SPECTROSCOPY

Near infrared spectroscopy is normally considered to be in the range of electromagnetic spectrum from 12,000 to 4000 cm^-1^ (Smith-Moritz et al., 2011). This wavelength region has two major advantages: first, the speed of spectral acquisition is high, which facilitates the real-time data collection for process control; secondly, the wide applicability to a diverse ranges of materials with little or no sample preparation (Schwanninger et al., 2011). This allows NIR to be effective for online monitoring and quality control of a wide variety of product properties and manufacturing processes (Workman, 2001; Kelley et al., 2004a; Tsuchikawa, 2007; Jin and Xu, 2011). Because of this, NIR has been extensively used as a high-throughput method to determine chemical, physical, mechanical, and fuel properties of woody biomass during the past 20 years.

However, there are some disadvantages to NIR. Although NIR absorption spectra have similar patterns to those in the mid-IR, they have wider separation, more anti-symmetry, and weaker intensity due to the fact that it is the combination and overtone bands from fundamental vibrations involved in NIR region. Therefore, the interpretation of NIR spectra are much harder than mid-IR (Schwanninger et al., 2011; Lupoi et al., 2014).

The utility of band assignments depends on the purpose of specific research or application. There is ongoing discussion around the necessity of interpreting NIR spectra in detail. Chemical/physical information contained in the NIR spectra can be used for detailed analysis (Schwanninger et al., 2011). However, it is not necessary to fully understand the chemical details for NIR to be useful for quantitative analysis. If NIR is used as a fast tool in distinguishing samples and in building prediction models for biomass properties, the detailed assignments are generally not needed. Statistical analysis for extracting useful information is essential for this purpose (Xu et al., 2013). Meaningful scientific insight of structural information could be better gained with the help of both statistical analysis and band assignments.

## NIR BAND ASSIGNMENT AND DATA PROCESSING

In NIR analysis, data points are usually collected in reflectance form (*R*) and converted to log_10_(1/*R*) form, which is equivalent to an absorbance spectrum.

As stated above, knowledge regarding band assignment is important for the understanding of chemical structures in biomass and there are several references on NIR band assignments (Tsuchikawa et al., 2003; Schwanninger et al., 2011; Via et al., 2013). Commonly assigned vibrations in the NIR spectra of woody biomass include (Schwanninger et al., 2011):

(1) 1370–1471 nm: First and second overtones of O–H stretching vibrations from free or weakly bonded O–H in carbohydrates and first overtones of C–H, C_aromatic_–H stretching vibrations, such as first overtone of O–H stretching in free OH group or OH group with a weak H-bond from cellulose, xylan, and glucomannan (1386, 1414, 1428, 1471, 1477–1484), first overtone of O–H stretching in phenolic hydroxyl groups from extractive or lignin (1410, 1447, 1448), first overtone of C–H stretching and bending in aromatic associated C–H from lignin (1417, 1440).
(2) 1471–1632 nm: First overtone of O–H stretching from strong O–H bonded group, semi-crystalline and crystalline region of cellulose (1473–1632) or intramolecular H-bond in glucomannan (1471, 1493).
(3) 1666–2000 nm: First overtone of aliphatic and aromatic C–H stretching vibrations and O–H combination bands from extractives/lignin (e.g., 1668, 1674, 1684, 1726), hemicellulose (e.g., 1720, 1724), cellulose (e.g., 1723, 1731), which are overlapped with each other and water band (e.g., 1887–2000).
(4) ABOVE 2000 nm: Assignment in this region is difficult due to high number of possibilities for the coupling of vibrations.

There are a number of well-established NIR spectra preprocessing techniques that can be used to achieve resolution enhancement and to more precisely locate band position. Methods for spectral data preprocessing include: (1) smoothing and derivatization (Denoyer and Dodd, 2002; Rousset et al., 2011) such as using the algorithm based method used by Savitzky and Golay (1964), (2) calculation of differential spectra (Rousset et al., 2011), and (3) Fourier self de-convolution, curve fitting (Ozaki et al., 2001) with more advanced techniques involving PCA (Fackler and Schwanninger, 2010) and two dimensional correlation analysis (Ozaki et al., 2001; Schwanninger et al., 2011).

Among those preprocessing methods, derivatives are widely used to reduce the impact of overlapping peaks and baseline variation. However, there is a concern that generating derivatives can possibly generate false information. Both the shape of the spectrum and the data processing algorithms have an impact on band shape and location. Differences between the location of the bands between the raw and the second derivative spectrum can be more than 20 cm^-1^ (5 nm). Researchers have also reported that the second derivative form was not always more precise than the normal form for the prediction of lignin in wood (Michell, 1995; Xu et al., 2013). Therefore, when spectral data is processed with the second derivative, possible peak shifts should be taken into consideration. The same consideration is also important for deriving conclusions from processing spectra of PCA and regression coefficients from PLS (Schwanninger et al., 2011).

## NIR SPECTROSCOPY COUPLED WITH PCA

The primary application of NIR coupled with PCA is to classify biomass samples of various origins or from different pretreatments without conducting laborious traditional wet chemistry techniques on all samples. Related areas of this application are summarized below:

(1) Related to species/plant fractions (Michell, 1995; Kelley et al., 2004a; Labbe et al., 2008a,b; Nkansah et al., 2010);
(2) Related to genetic engineering of feedstock crops (Baillères et al., 2002; Sandak and Sandak, 2011; Zhou et al., 2011);
(3) Related to chemical/thermal/biological treatments (Kelley et al., 2004b; Yang et al., 2007; Houghton et al., 2009; Krongtaew et al., 2010).

**Figure 1:**
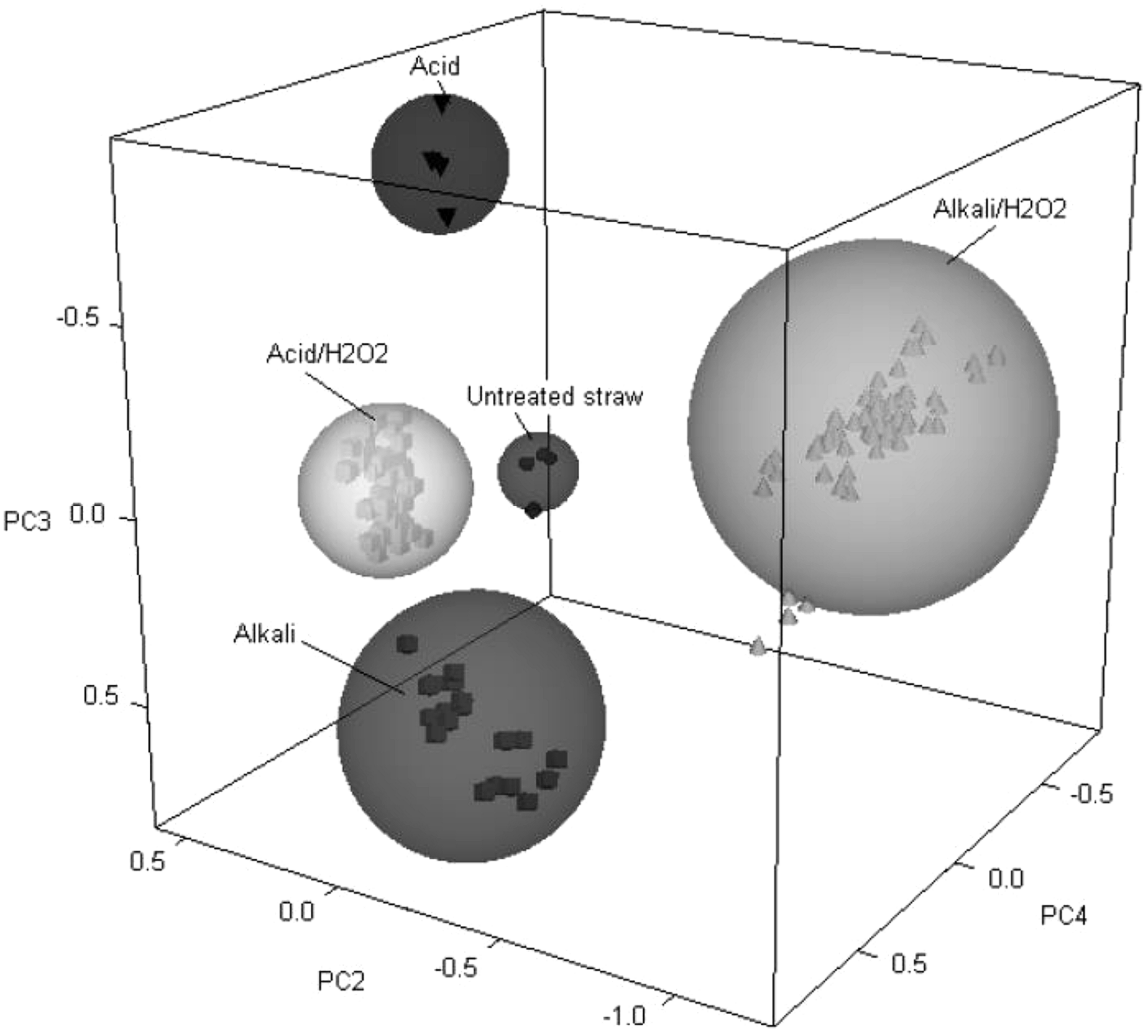
**PCA scores plot of untreated wheat straw samples (•) and samples treated with acid (▼), alkali (▪), acid/H_**2**_O_**2**_ (□), and alkali/H_**2**_O_**2**_ (Δ) as reproduced from literature (Krongtaew et al., 2010)**.

For example, in order to evaluate the impact of biomass pretreatments (including acid and alkaline pretreatments, some in combination with hydrogen peroxide) on the change of cell wall compositions of wheat and oat straw, FT-NIR was utilized to characterize raw and pretreated straw (Krongtaew et al., 2010). Second derivatives from NIR absorption bands were generated and evaluated to show the changes in properties related to biomass recalcitrance during subsequent bioethanol production. These properties include the change of lignin, hemicelluloses; as well as amorphous, semi-crystalline, and crystalline regions of cellulose moieties of pretreated sample. PCA of derivative data was efficiently utilized to differentiate the alterations in chemical structure of straw due to different pretreatment methods as shown in **Figure 1**. It was demonstrated that FT-NIR coupled with PCA is a powerful tool to assess biomass digestibility, with a potential to be used in process control in the area of biomass utilization or energy conversion.

## NIR SPECTROSCOPY COUPLED WITH PLS

One of the main applications of NIR coupled with PLS is to build regression models for the prediction of biomass properties, such as lignin content, S/G-lignin ratio, moisture content, heating value (Kelley et al., 2004a; Rousset et al., 2011; Schwanninger et al., 2011).

Related areas of the application of NIR coupled with PLS in existing literatures are summarized below:

(1) Prediction of cell wall components (Michell, 1995; Sanderson et al., 1996; Tucker et al., 2001; Baillères et al., 2002; Kelley et al., 2004a; Lovett et al., 2004; Yeh et al., 2004; Jin and Chen, 2007; Labbe et al., 2008b; Philip Ye et al., 2008; Wolfrum and Sluiter, 2009; Nkansah et al., 2010; Hou and Li, 2011; Sandak and Sandak, 2011; Smith-Moritz et al., 2011; Zhou et al., 2011).

**Figure 2:**
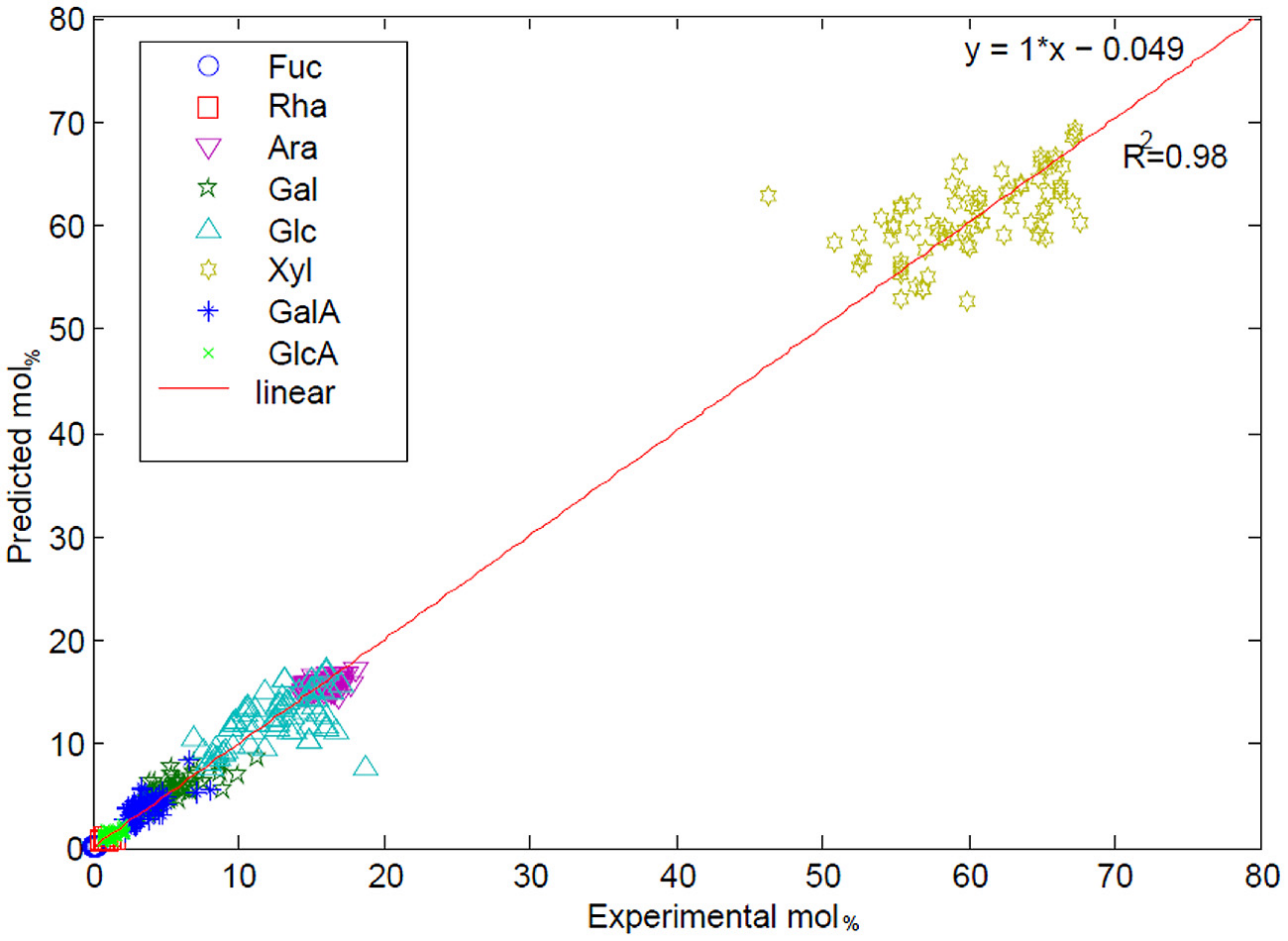
**A correlation analysis predicted (PLS model of FT-NIR) versus experimentally determined monosaccharide composition (mol%) of rice leaf samples.** The correlation coefficient between experimental and predicted values was calculated to be *R*^2^ = 0.98 as reproduced from literature (Agblevor et al., 1994; Smith-Moritz et al., 2011).

For example, in order to identify specific monosaccharide outliers from a plant mutant population, FT-NIR coupled with PLS regression was utilized to analyze plant leaves of *Arabidopsis* (Smith-Moritz et al., 2011). Various Arabidopsis cell wall mutants were analyzed for prediction model building. PCA was performed on pre-processed and area-normalized NIR spectra, followed by calculation of the Mahalanobis distance, a linear discriminate analysis technique to identify outliers using PCA results. By using this technique, a pilot study was conducted which consisted of 550 mutant lines (3590 leaf samples), resulting in a set of 235 leaf samples as Mahalanobis outliers. Quantitative information about monosaccharide composition is gained by means of PLS modeling with known biochemical values and FT-NIR spectra. The correlation between predicted and experiment determined monosaccharide composition (mol%) of 226 rice leaf samples are shown in **Figure 2** with *R*^2^ = 0.98 (Smith-Moritz et al., 2011).

(2) Prediction of other physical properties (Thygesen, 1994; Hoffmeyer and Pedersen, 1995), mechanical properties (Kelley et al., 2004a; André et al., 2006), fuel properties (Lestander and Rhen, 2005; Labbe et al., 2008a).

For example, NIR coupled with PLS has been used to predict cell wall chemistry and mechanical properties of loblolly pine from different radial locations and heights of trees grown in Arkansas (Kelley et al., 2004a). Mechanical properties include three point bending test and related microfibril angle. The correlation between experimental data and predicted data from PLS modeling is very strong with correlation coefficients (*r*) as high as 0.80. A reduced spectral range (650–1150 nm) usually available in handheld NIR spectrometers was also demonstrated to be useful for predicting mechanical properties.

## BIOMASS CHARACTERIZATION BY Py-mbms

Py-mbms has been intensively used for studies of biological and synthetic macromolecules, such as wood, grasses, carbon in soil and chars. It has proved to be an efficient and powerful analytical tool (Evans and Milne, 1987; Kelley et al., 2002; Labbe et al., 2005; Magrini et al., 2007; Sykes et al., 2008; Mann et al., 2009; French and Czernik, 2010). Detailed description of this technology is available in the above references. In short, the Py-mbms is composed of a pyrolysis furnace and a free-jet mbms. Typically the furnace is preheated to 500°C before ground sample of biomass is inserted into the inert atmosphere of the furnace. Pyrolysis products from biomass in the furnace are swept out of the furnace into the mbms by an argon gas stream. Molecular fragments contained in the pyrolysis vapor are expanded in a series of vacuum chambers to be quenched; so that intermolecular collisions are prevented. A low-energy electron beam (17–23 eV) in the triple quadruple mass spectrometer is employed to produce a positive ion mass spectrum. The positive ion stream is magnified and collected by the detector.

Mass peaks were assigned to chemical fragments produced from fast pyrolysis of biomass for direct interpretation (Evans and Milne, 1987). The spectra from Py-mbms is also interpreted with the help of MVA tools, especially PLS and PCA (Hoover et al., 2002; Kelley et al., 2002, 2004b; Labbe et al., 2005; Magrini et al., 2007; Mann et al., 2009).

## Py-mbms PEAK ASSIGNMENT AND DATA PROCESSING

During data acquisition of Py-mbms, amplified positive ions from biomass pyrolysis vapor are scanned continuously; then the signal is collected by a computer. Approximate evolution time of fast pyrolysis for a sample of 4 mg is less than 1 min. During the evolution time there are typically 50 single scans collected. Biomass with larger sample size will need longer evolution time and more scans during fast pyrolysis. Together with single scan spectrum, time resolved profile and averaged spectrum can be collected by the computer acquisition software (Evans and Milne, 1987).

**Figure 3:**
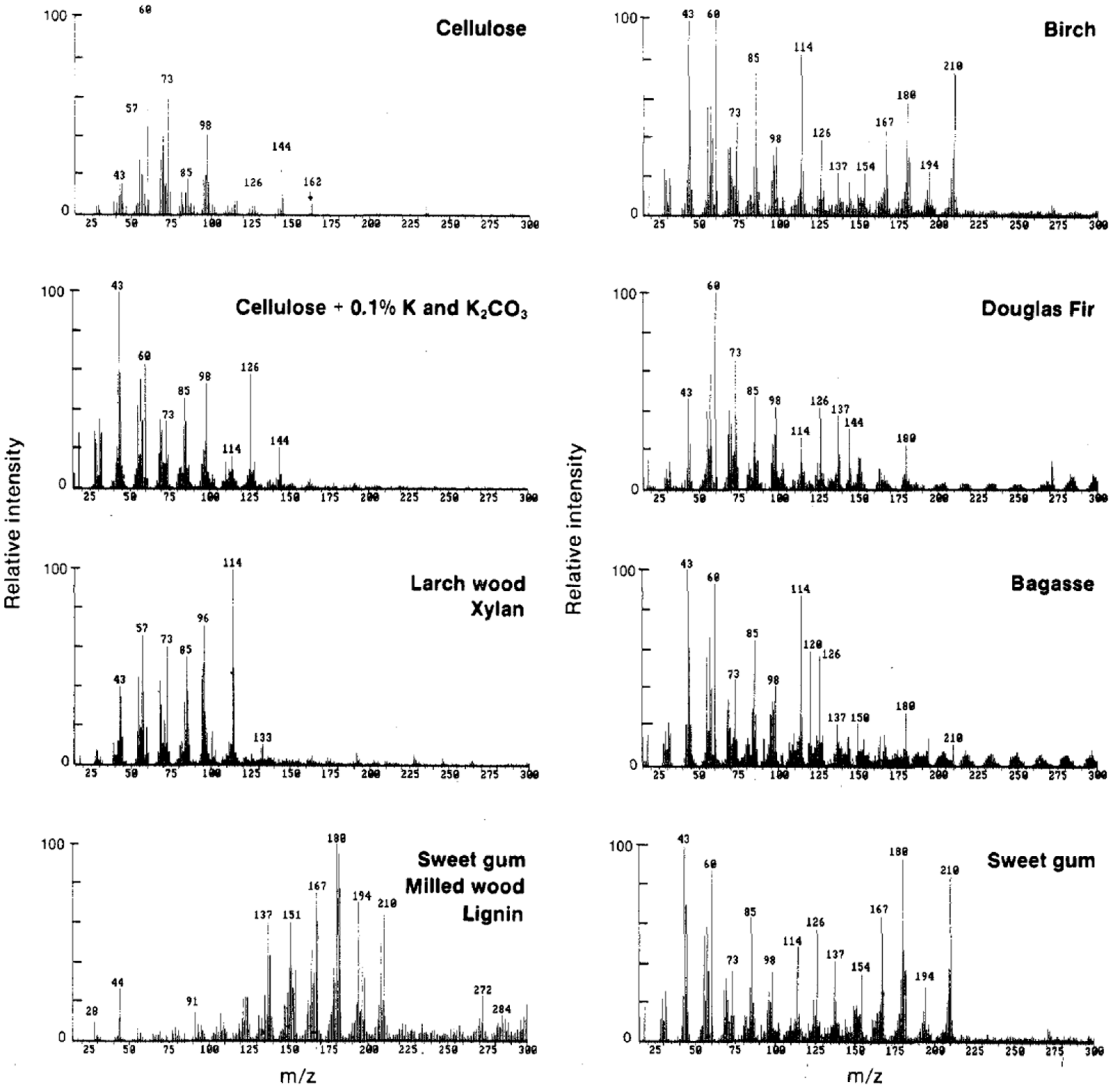
**Characteristic mass spectral patterns of primary pyrolysis products for several whole biomass samples and for separated constituents of biomass (Evans and Milne, 1987)**.

Average spectra are also known as spectral “fingerprints.” Spectral fingerprints gained at analytical pyrolysis temperature of 500–550°C and the molecular beam free jet expansion represent primary products from biomass pyrolysis. Studies shown that at this temperature range, molecular structure of the original biomass is well preserved and there is no interaction observed among organic components during pyrolysis, although inorganics may alter the pyrolysis pathways of the carbohydrates (Evans and Milne, 1987). Thus, with known peak assignment, spectral “fingerprints” generated could be used to depict the molecular structure of chemical composition in biomass. A summary of important peak assignment in biomass is shown in **Table 1** (Evans and Milne, 1987; Sykes et al., 2008). Characteristic spectral fingerprints of whole biomass samples and separated constituents of biomass are shown in **Figure 3** (Evans and Milne, 1987).

**Table 1:** Peak assignments associated with Py-mbms spectrum for *Populus* wood based on literature (Evans and Milne, 1987; Sykes et al., 2008).

Mass peaks (*m*/*z*)	Assigned products	S or G precursor
57, 73, 85, 96, 114	From C5 sugar	
57, 60, 73, 98, 126, 144	From C6 sugar	
94	Phenol, dimethylcyclopentene	
108	Methyl phenol (*o*-cresol, *m/p*-cresol)	
110	Dihydroxybenzene, 5-methylfurfural	
120	Vinylphenol	
122	Ethylphenol, ethylphenol, benzoic acid	
124	Guaiacol (2-methoxyphenol), trimethylcyclopentenone	G
137^*^	Ethylguaiacol, homovanillin, coniferyl alcohol	G
138	Methylguaiacol	G
150	*p*-Inylguaiacol, coumaryl alcohol	G
152	4-Ethylguaiacol, vanillin	G
154	Syringol (2,6-dimethoxyphenol)	S
164	Isoeugenenol, eugenol	G
167^*^	Ethylsyringol, syrinylacetone, propiosyringone	S
168	4-Methyl-2,6-dimethoxyphenol	S
178	Coniferyl aldehyde	G
180	Coniferyl alcohol, syringylethene	S, G
182	Syringaldehyde	S
194	4-Propenylsyringol	S
208	Synapyl aldehyde	S
210	Synapyl alcohol	S

* Fragmention.

*m/z: mass to charge ratio.*

* S, syringyl lignin; G, guaiacol lignin.*

Pyrolysis-molecular beam mass spectrometry has been successfully applied in many biomass-related studies, including the research of cellulose, cellulose with inorganics, many woods, xylan, milled wood lignin, bagasse (Evans and Milne, 1987), herbaceous biomass under different storage environments (Agblevor et al., 1994), hardwood sawdust and its torrefaction products (Nimlos et al., 2003), and poplar grown under different nitrogen conditions (Sykes et al., 2009).

For example, in the study of bark phenolysis conducted by Alma and Kelley, bark and its phenolysis products from Calabrian pine, Lebanon cedar, acacia, and European chestnut were characterized using Py-mbms (Alma and Kelley, 2002). From the results of Py-mbms averaged spectra, it was shown that bark (1) has less common lignin peaks at *m*/*z* 180, 194, 210 assigned to coniferyl alcohol/vinylsyringol, 4-propenylsyringol/ferulic acid, and sinapyl alcohol, respectively; (2) has unique triplet of peaks at *m*/*z* of 96, 97, 98 assigned to furans; and (3) has more phenols, such as peaks at *m*/*z* of 110, 124, 150, and 164 assigned to catechol, guaiacol, vinyl guaiacol, and isoeugenol. In softwood bark, extractives and lignin dimers can be identified at *m*/*z* of 298, 300, 302, and 272 assigned to didehydroabeitic acid, dehydroabeitic acid, abeiticacid, and lignin dimer, respectively (Alma and Kelley, 2002). These results are consistent with known differences between bark and wood.

### SELECTED PEAKS FROM Py-mbms RAW DATA

As summarized above, certain Py-mbms peaks can be unambiguously assigned to specific biomass components. Lignin fragments are particularly easy to identify. Because of this, Klason lignin content of biomass can be directly estimated from Py-mbms spectral fingerprints. Firstly, spectral fingerprints of samples are area/mean normalized for the mass of the original sample. Then, the total intensity of lignin related peaks from the normalized spectrum is calculated. After that, a correction factor is calculated by dividing the known Klason lignin value by the summed intensity of a NIST standard material. The correction factor can be used to convert the total intensity of lignin related peaks to Klason lignin content (Davis and Lagutaris, 2002; Sykes et al., 2008, 2009; Ziebell et al., 2013). Similarly, S/G ratios were determined by dividing the sum of S-lignin peaks by the sum of G-lignin peaks excluding peaks associated with both S and G fragments (Davis and Lagutaris, 2002; Sykes et al., 2008, 2009; Mann et al., 2009; Ziebell et al., 2013).

For example, corrected lignin values and S/G-lignin ratio were determined from Py-mbms for 800 greenhouse-grown poplar trees grown under atmosphere containing different amount of nitrogen (Sykes et al., 2009). Lignin contents ranged from 13 to 28% whereas S/G ranged from 0.5 to 1.5. It was shown that the variations in cell wall composition were larger in the plants grown under high nitrogen conditions than those grown under low nitrogen conditions.

Similarly, “within-tree” variability in lignin content and S/G ratio with increasing height and increasing ring for poplars was determined by Py-mbms (Sykes et al., 2008). Wood disks from seven different poplar trees, which were seven years old, were sampled at five different heights of 0.3, 0.6, 1.2, 1.8, and 2.4 m from base to stem. Samples were collected from the north side of each wood disk taken at height of 1.2 m to study difference between growth rings. According to results from Py-mbms, ring effect on lignin content was significant while the effect of height was small. Higher S/G ratio was observed with increasing ring size, whereas lignin content decreased. S/G ratio was determined for switchgrass grown under different environment using the same methodology (Mann et al., 2009).

## Py-mbms COUPLED WITH PCA

Pyrolysis-molecular beam mass spectrometry coupled with PCA provides a fast analytical method to distinguish a large number of biomass samples. It has been used to study biomass compositional variations due to species (Evans and Milne, 1987; Agblevor et al., 1994; Alma and Kelley, 2002; Kelley et al., 2004b), genetic engineering (Labbe et al., 2005; Davis et al., 2006), different growth environments (Mann et al., 2009; Sykes et al., 2009), thermal (Nimlos et al., 2003)/chemical (Alma and Kelley, 2002; Kelley et al., 2004b)/biological (Kelley et al., 2002; Arantes et al., 2009) treatments, and various storage/collection (Agblevor et al., 1994) methods.

**Figure 4:**
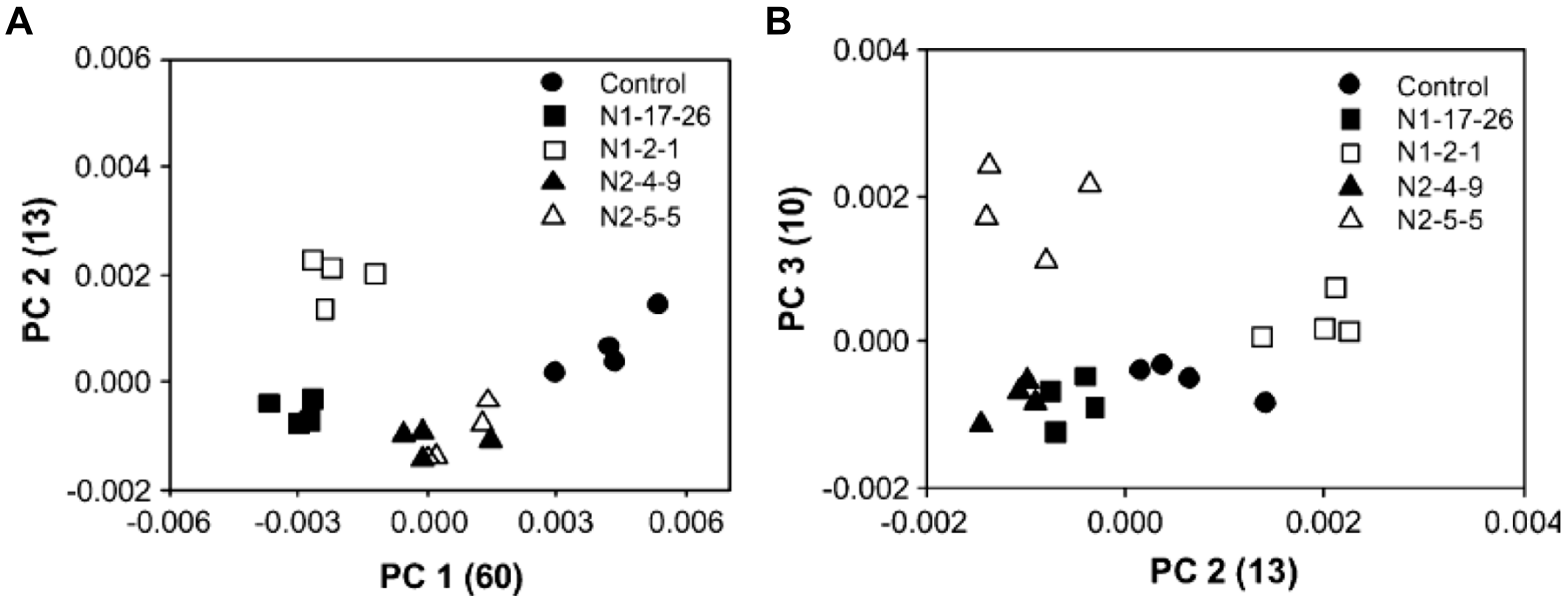
**Scores plot of PCA of Py-mbms data for original and transgenic aspens; **(A)** PC1 versus PC2; **(B)** PC2 versus PC3; N1 samples are clearly separated from control samples in **(A)** while two N2 samples are not distinguishable; in **(B)** two N2 samples are clearly separated by PC3 as reproduced from literature (Labbe et al., 2005)**.

**Figure 5:**
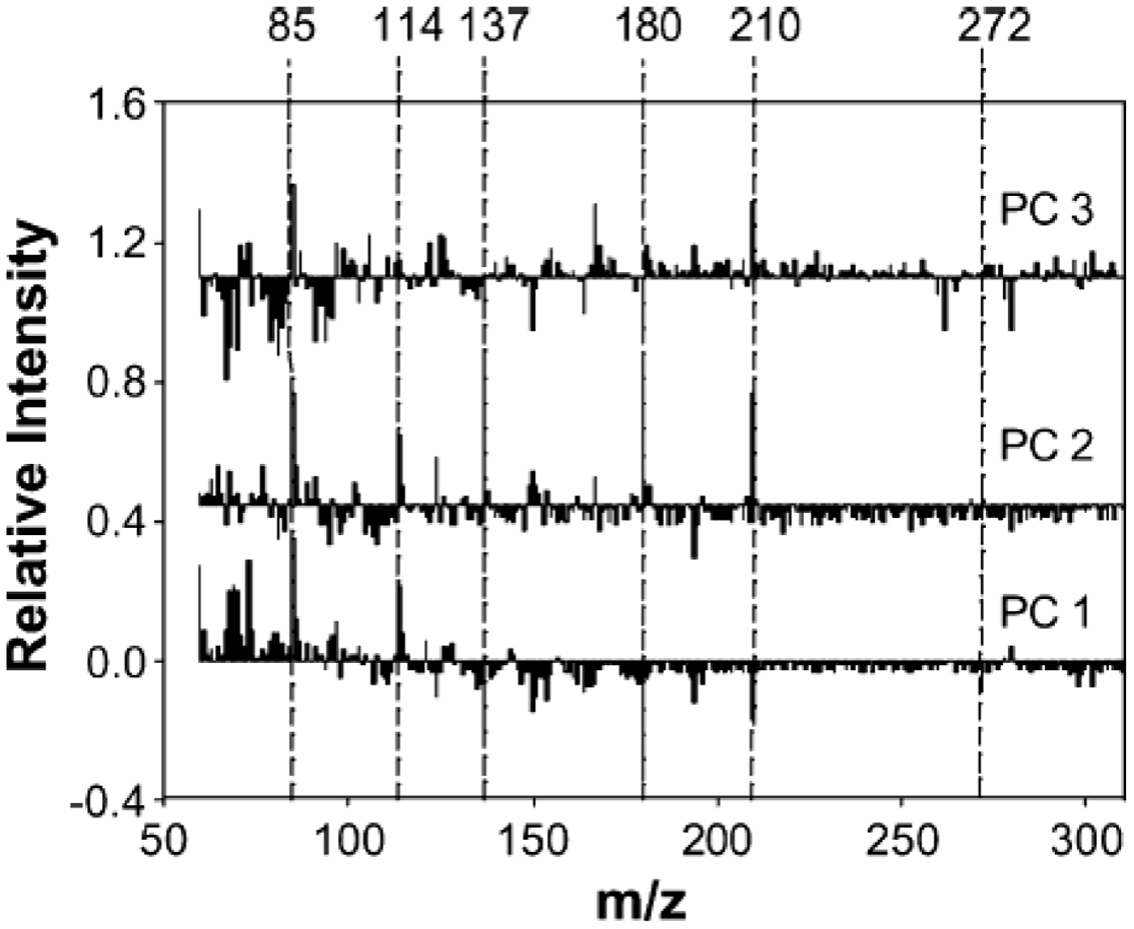
**Loadings from PCA of Py-mbms data for original and transgenic aspens; from top to bottom: PC3, PC2, PC1; C5 carbohydrates (*m*/*z* 85 and 114) and lignin (*m*/*z* 137, 180, 210, and 272) are highlighted for PC1 as reproduced from literature (Labbe et al., 2005)**.

For example, Py-mbms coupled with PCA has been used to measure the overall composition between and within a series of original and transgenic aspens (Labbe et al., 2005). Two clones were transformed with GRP-*iaa*M gene (N1-17-26 and N1-2-1) and GRP-*iaa*M/35S-ACCase (N2-4-9 and N2-5-5). PCA analysis was conducted for data analysis with an attempt to identify chemical differences between the modified and control aspens. **Figure 4** shows PCA scores plots with four replicate samples from five different aspen samples. **Figure 4A** shows a plot of PC1 versus PC2, while **Figure 4B** shows a plot of PC2 versus PC3. In **Figure 4A**, there is clear separation between the two N1 samples while two N2 samples are indistinguishable. Moreover, two N2 samples are clearly separated from each other along PC3 as shown in **Figure 4B**. The loadings from PCA are shown in **Figure 5**. Using PC1 loadings as an example, C5 carbohydrates (*m*/*z* 85 and 114) and lignin (*m*/*z* 137, 180, 210, and 272) are highlighted for PC1. This suggests there are more C5 sugars and less lignin in controls than those in N1 and N2 samples (Labbe et al., 2005).

Pyrolysis-molecular beam mass spectrometry had been also used to study the impact of storage environment on herbaceous material. Weathered and unweathered fractions of three types of herbaceous biomass after storage at 18 different conditions for 6–9 months were analyzed by Py-mbms coupled with PCA (Agblevor et al., 1994). Two major trends in the data were shown by PCA (factor analysis): major clusters were distinguished by relative nitrogen contents between switchgrass and the other two herbaceous biomass samples; subgroups of weathered and unweathered materials are clearly separated as subgroups within the major clusters. According to the variance diagram (similar to loadings plot), lower amount of carbohydrates constituted the major chemical difference between weathered and unweathered samples (Agblevor et al., 1994). This observation is consistent with results from traditional wet chemical analysis and Py-GC/MS.

In some cases, there is no separation of clusters in PCA scores plot. This indicates that there is no comprehensive difference among samples for the specific chemical features included in those particular PCs.

For example, three transgenic clones of populous wood were analyzed by Py-mbms, GC/MS, and traditional wet chemical techniques to screen for possible variations in cell wall composition due to genetic engineering (Davis et al., 2006). Various *Bacillus thuringiensis* (Bt) gene-containing constructs were used to transform poplar genotypes. Transgenic poplar was then compared with non-transgenic control. PCA results showed that there were generally no distinct groupings of individual transgenic lines or non-transgenic controls, indicating no significant differences in cell wall composition between control and transgenic poplars (Davis et al., 2006).

## Py-mbms COUPLED WITH PLS

One of the primary applications of Py-mbms has been the development of prediction models for biomass compositional properties. Results from conventional methods of cell wall compositional analysis were used as references to build calibration models with capability for predicting the composition for future samples. As a result, laborious wet chemistry techniques can be eliminated. PLS regression is widely used in this arena for both woody (Tuskan et al., 1999; Labbe et al., 2005) and herbaceous biomass (Agblevor et al., 1994; Kelley et al., 2004b; Mann et al., 2009).

For example, the effectiveness of NIR and Py-mbms in predicting cell wall composition of various agricultural residues was tested (Kelley et al., 2004b). Forty-one samples from 14 species with known content of lignin and six individual sugars were analyzed by NIR and Py-mbms. Prediction models were built between spectral data from both techniques and cell wall compositional data. Correlation coefficient and root mean square error data for each calibration and validation model was presented and compared. Good correlations between the predicted and measured value of major components (lignin, glucose, xylose, and mannose) were obtained (correlation coefficients of both calibration and validation model are above 0.80 for both NIR and Py-mbms), while correlations for minor sugars (mannose, galactose, arabinose, and rhamnose) were not as good. A summary of PLS prediction of chemical composition from Py-mbms is presented in Table 2. According to the author, more samples for specific feedstocks are needed for building improved models. This work also did a thorough comparison between NIR and Py-mbms (Kelley et al., 2004b).

**Table 2:** Summary of the PLS-2 predictions of chemical composition from Py-mbms (six PCs; Kelley et al., 2004b).

	Lignin	Glucose	Xylose	Mannose	Galactose	Arabinose	Rhamnose
r(CALB)	0.85	0.85	0.87	0.92	0.83	0.70	0.80
r(VALD)	0.77	0.75	0.81	0.86	0.65	0.54	0.71
RMSEC	4.60	6.20	3.40	1.40	0.40	0.50	0.10
RMSEP	5.50	8.00	4.10	1.80	0.50	0.60	0.10

Other than being used to predict cell wall composition of biomass, PLS has been applied in predicting other biomass properties and processing parameters. The acidic phenolysis condition of bark (Alma and Kelley, 2002), weight loss during fungal degradation of spruce (Kelley et al., 2002) and carbon content/fraction of different soils (Hoover et al., 2002; Magrini et al., 2007) were also predicted by Py-mbms coupled with PLS.

For example, NIR and Py-mbms were utilized to monitor the chemical changes of wood undergoing brown-rot degradation. In this case, spruce blocks were infected by *Postia placenta* or *Glaoeophyllum trabeum* for 0, 2, 4, 8, and 16 weeks (Kelley et al., 2002). Weight losses over the time period were monitored and recorded. PLS models were built to predict weight loss. Strong correlation between recorded weight loss and predicted weight were obtained (correlation coefficients of calibration model reached 0.98, while those of test model reached 0.96 for both NIR and Py-mbms). The regression coefficients for PLS model from Py-mbms data show that weight loss during decay is positively correlated to carbohydrates (*m*/*z* 85, 114, and 126) and negatively correlated to monomethoxylated lignin fragments (*m*/*z* 123, 138, and 151; Kelley et al., 2002).

## CONCLUSION

Compared to traditional techniques in biomass characterization, high-throughput analytical techniques, such as NIR and Py-mbms have been proved to be efficient tools in exploring the chemical features of different biomass samples with minimal sample preparation. These high-throughput techniques coupled with MVA have been demonstrated to be efficient in identifying outliers, comparing samples (using PCA), and building prediction models (using PLS). Both NIR and Py-mbms coupled with MVA could be used not only for characterizing the cell wall chemistry, but also for predicting other chemical, physical, mechanical, and fuel properties. In comparison with Py-mbms, NIR has the advantages of low cost and simple instrumentation, field-portable, and nondestructive, whereas Py-mbms provides superior information of molecular structural information.

Thus, we recommend that NIR and Py-mbms coupled with MVA should be widely employed for biomass characterization. Additional fundamental work on assigning NIR vibrations band and Py-mbm speaks for modified biomass or biomass related products are recommended since current assignment are mainly based on the study of unmodified biomass. Lack of assignments for new bands/peaks in modified biomass limit the application of these two techniques in exploring the fundamental changes of chemical composition of modified biomass. Also, comparison and correlation between analytical results from Py-GC/MS and Py-mbms should be encouraged because of the important similarity and differences in these two techniques are critical for using those techniques for the characterization of biomass molecular structure.

## Conflict of Interest Statement 

The authors declare that the research was conducted in the absence of any commercial or financial relationships that could be construed as a potential conflict of interest.
